# Cardiovascular risk and obesity

**DOI:** 10.1186/s13098-019-0468-0

**Published:** 2019-08-28

**Authors:** C. Cercato, F. A. Fonseca

**Affiliations:** 1 Grupo de Obesidade e Síndrome Metabólica, Hospital das Clínicas, Faculdade de Medicina, Universidade de São Paulo, São Paulo, Brazil; 2 Escola Paulista de Medicina, Universidade Federal de São Paulo, Rua Loefgren 1350, São Paulo, SP CEP 04040-001 Brazil

**Keywords:** Obesity, Cardiovascular risk, Clinical assessment

## Abstract

**Background:**

This is an overview of the mechanisms of obesity and its relation to cardiovascular risks, describing the available treatment options to manage this condition.

**Main body:**

The pathogenesis of obesity includes the balance between calories consumed and energy expenditure followed by the maintenance of body weight. Diet, physical activity, environmental, behavioral and physiological factors are part of the complex process of weight loss, since there are several hormones and peptides involved in regulation of appetite, eating behavior and energy expenditure. The cardiovascular complications associated to obesity are also driven by processes involving hormones and peptides and which include inflammation, insulin resistance, endothelial dysfunction, coronary calcification, activation of coagulation, renin angiotensin or the sympathetic nervous systems. Pharmacological treatments are often needed to insure weight loss and weight maintenance as adjuncts to diet and physical activity in people with obesity and overweight patients.

**Conclusion:**

To accomplish satisfactory goals, patients and physicians seek for weight loss, weight maintenance and improvement of the risk factors associated to this condition, especially cardiovascular risk.

## Background

Obesity, generally defined as an excess of body-fat mass, is a known global epidemic that can have very serious consequences like increased risk of morbidity and reduced life expectancy [1–3].

The World Health Organization (WHO) estimated that in 2016 more than 1.9 billion adults were overweight (39% of the population) and over 650 million (13% of the population) were people with obesity [4].

Even though the development of obesity is multifactorial with genetic, environmental and lifestyle causes, it is extensively associated with comorbidities such as cardiovascular diseases, diabetes, hypertension, cancer, and sleep disorders [2, 3, 5–8].

This is an overview of the mechanisms of obesity and its relation to cardiovascular risks, describing the available treatment options to manage this condition.

## Disease pathogenesis

The pathogenesis of obesity is influenced by the balance between calories consumed and energy expenditure followed by the reset of body weight [1, 3, 5]. However, this is not as simple as an equation, and there are secondary processes that contribute to this complex condition.

In addition to diet, environmental and behavioral factors enhance the risk for obesity. Obesity pathogenesis is not only about how excess body fat is acquired, but also about how this excess is biologically assimilated [1, 8–10]. Several metabolic parameters (glucose, insulin, fatty acids, adipocytes, gut microbiome) are involved in the obesity pathogenesis, as well as all the systems (gastric, nervous) that regulate appetite control or food intake [1, 3, 5]. Genetic factors and age are also parameters that can modulate the phenotypic expression of obesity [5, 11].

The most important systems regulating body weight and appetite are the adipose tissue, gastrointestinal hormones and nervous system, which receive signals and, in response, generate the appropriate stimuli [5]. Gastric distension is a signal for satiety, gastric emptying are signals for hunger, while nutrients, neural impulses and hormones act as signals in the regulation of energy intake and expenditure. Likewise, glucose refers to a sensation of satiety whereas its decrease promotes hunger. As for the nervous system, the peripheral nervous system acts by stimulating the thermogenic tissues, while the sympathetic nervous system maintains the energy expenditure [3, 5].

The most important hormones that are involved in the control of hunger and satiety signals are leptin, insulin, cholecystokinin (CCK), glucagon-like peptide-1 (GLP-1), peptide YY (PYY), and ghrelin. These act by transmitting information about the energy status to hypothalamus and brain cells, which interact with the reward system influencing the need to eat [3].

Leptin is an anorexigenic hormone produced proportionally from the adipose tissue that regulates the lipid metabolism by crossing the blood–brain barrier via a saturable transport system and communicating the energy status to the hypothalamus, and so down-regulating the appetite stimulators and up-regulating the anorexigenic alpha-melanocyte-stimulating hormone that reduces food intake [3, 5, 12]. Leptin is an anti-obesity hormone, its concentration in the blood is elevated in people with obesity, however individuals become resistant to its satiety and weight-reducing effect [3, 13–15]. Also, leptin is known for its sympathetic nerve system activation, renal hemodynamics, blood vessel tone, and modulation of blood pressure [15].

Insulin is a pancreatic hormone that regulates the level of blood glucose. After meals, glucose levels increase and secretion of insulin is activated. Insulin binds to the hypothalamus receptors to decrease food intake, however insulin is sensitive to its concentration levels, which vary with the amount of adipose tissue and fat. Similarly to leptin, insulin resistance may occur in obesity as a consequence of complex mechanisms. Insulin modulates the reward system pathways by inhibiting its circuits which are associated to eating behaviors [3].

Ghrelin is a gut peptide growth hormone with orexigenic action that acts on the hypothalamus receptors to exert metabolic effects by inhibiting insulin secretion and regulating gluconeogenesis and glycogenolysis. It’s a fast-acting hormone, or initiator of feeding, since its levels increase prior to food ingestion. Ghrelin’s signaling decreases thermogenesis and regulate energy expenditure, promoting adiposity [3, 5]. Additionally, this hormone has other mechanisms which interplays with the energy equilibrium of other systems playing important roles in areas like cardioprotection, muscle atrophy, bone metabolism or cancer [16].

Peptide YY is a small gut peptide which responds to feeding and acts through the anorexinergic cycle on the hypothalamus to reduce intestinal motility, gallbladder and gastric emptying and thus decreasing the appetite and increasing satiety [3].

Glucagon-like peptide-1 is a gut hormone, co-released with PYY after meals. Its main functions are to stimulate insulin secretion, to increase β-cell growth and survival, to prevent glucagon release and to suppress appetite. The physiological effects of GLP-1 are mediated through its receptor GLP-1R, expressed in pancreatic cells, heart, kidney, stomach, intestine, pituitary gland and hypothalamus. Its stimulation increases intracellular calcium levels, adenylate cyclase activity, and promotes the activation of several signaling pathways [17]. It has been demonstrated that GLP-1 acts as cardiovascular protector by inhibiting thrombosis, preventing atherogenesis, protecting against vascular inflammation and oxidative stress [3, 18].

Cholecystokinin is a gut peptide hormone and a brain neuropeptide responsible for stimulating the digestion, delaying gastric emptying, promoting intestinal motility, enhancing stimulation of pancreatic digestive enzymes and bile from the gallbladder, and therefore controlling appetite [3].

These hormones and peptides regulate appetite, eating behaviors and energy expenditure by signaling on hypothalamus and brain cells which modulates the dopamine pathways [3].

In recent years, the role of intestinal microbiota with the development of obesity has been recognized with most bacteria belonging to the *phyla Bacteriodetes* or *Firmicutes*. Microbiota can be modified by diet and when obesity is induced in both murine models or humans, a predominance of *Firmicutes* has been reported in some studies [19–21]. The composition of the *phylum Bacteriodetes* are gram negative bacteria, which contain lipopolysaccharide (LPS). The release of LPS activates toll-like receptor 4, which elicits a pro-inflammatory pathway [22]. The increase of circulating LPS in obese individuals with predominant bacteria belonging to the Firmicutes phylum is not a paradox and can be explained by increased intestinal permeability [22].

## Cardiovascular risk

It is well known that obesity is an independent risk factor for cardiovascular disease (CVD) and one of the main causes of the increased risk of diseases such as dyslipidemia, insulin resistance, high blood pressure (HBP) or hypertension, and atherosclerosis both in adults and children [23, 24].

Obesity and increased adipose tissue influence the pathogenesis of atherosclerosis. The adipose tissue, which is in fact a dynamic organ, is divided in white adipose tissue (WAT) and brown adipose tissue (BAT) and is associated with metabolic and inflammatory systems, with protective effects on energy homeostasis. WAT secretes peptides and proteins that act by regulating biological and physiological conditions and play an important role in obesity, insulin resistance, inflammatory and immune functions, atherosclerosis and cardiovascular disease [12–14, 25–30].

Adiponectin is a peptide produced in adipose tissue, that is expressed at high levels by lean, healthy people and becomes dysregulated in obesity [12, 26, 27].

Obesity is considered a state of inflammation with increased adipose tissue and decrease in adiponectin levels, which limits its ability to inhibit the inflammatory processes, perpetuating the inflammatory condition. This adipocyte dysregulations are contributing factors to the imbalance of body homeostasis and pro- and anti-inflammatory mechanisms, which contribute to obesity‐induced metabolic complications and vascular breakdown leading to cardiometabolic alterations [25, 26, 30–33]. In parallel to the development of obesity, inflammatory cell infiltrate occurs, not only in the adipose tissue, but in the pancreas and other tissues [34]. This inflammatory state can be detected early among adolescents with metabolic syndrome [35], and a clear relationship has been established between inflammatory biomarkers and cardiovascular events [36, 37].

**Fig. 1:**
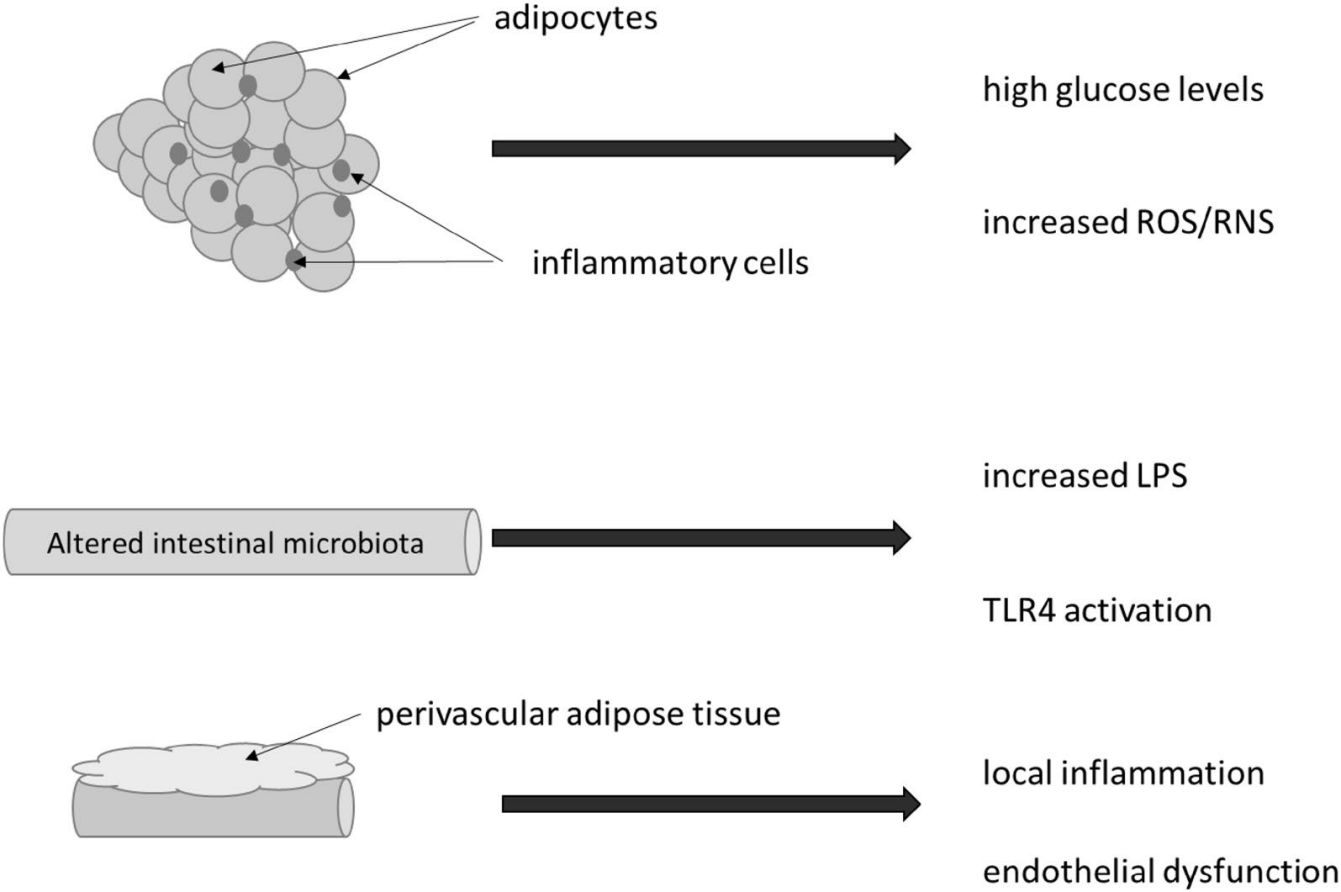
Process of inflammation in obesity. Inflammatory infiltrate into adipocyte cells is a common finding in subjects with obesity or metabolic syndrome and an inflammatory status can be detected by circulating biomarkers. In addition, an increased in the amount of reactive oxygen species (ROS) and reactive nitrogen species (RNS) can also be detected. In parallel, disturbances in the microbiota are related to increased lipopolysaccharide (LPS) release in the bloodstream which activates toll like receptor 4 (TLR4). Finally, increased perivascular adipose tissue promotes local inflammation and impairment of endothelium function

Additionally, obesity leads to insulin resistance and endothelial dysfunction due to the formation of metabolic products derived from lipids, hormones and proinflammatory cytokines. Endothelial dysfunction is associated with cardiovascular conditions, like atherosclerosis, hypertension, hyperlipidemia, and insulin resistance, which alters the insulin signaling pathway. Adiponectin can modulate the expression of endothelial cells affecting the key mechanisms involved in atherogenesis (stimulation of nitric oxide production, mitigation of pro-atherogenic mediators, coronary plaque stabilization, arterial vasodilation), thus acting as a protective factor for cardiovascular disease and increases insulin sensitivity [2, 12, 13, 30, 38]. Also, perivascular adipose tissue, particularly from obese individuals seems to promote local inflammation and impairment of endothelial function, thus providing a link between adipose tissue and vascular disease [39] (Fig. 1). More recently, the use of computed tomography has been proposed to evaluate the characteristics of perivascular adipose tissue composition which appears to identify patients with stable coronary disease at increased risk for acute coronary syndromes [40].

Development of low-grade inflammation by dysfunction of the adipose tissue diminishes its homeostatic protective effect causing adipocytes to produce inflammatory cytokines and extracellular proteins, that support infiltration and activation of immune cells. Immune cells that infiltrate dysfunctional adipose tissue are the key drivers for inflammation. This mechanism of adipose tissue infiltration is associated with the reduction of insulin sensitivity and glucose tolerance especially important in the process of regulating insulin resistance in type 2 diabetes (T2D), obesity and hypertension or atherosclerosis [32, 41–43]. Obesity is frequently associated with high glucose levels and endoplasmic reticulum stress with increased release of reactive oxygen and reactive nitrogen species, impairing insulin secretion, and insulin sensitivity [34]. On this context, the relevance of the c-Jun-N-terminal-kinase (JNK) as a pivotal role in the stress cell response has been highlighted [44].

Coronary calcification is a result of the atherosclerotic inflammation process, which is associated to obesity. Even though there is inconsistent evidence, there are studies to support the association of obesity and coronary calcification in adults at low risk for CVD, including the Framingham Study. In low-risk CVD patients, the distribution of body fat plays an important role in coronary atherogenesis, since it has been shown that a greater accumulation of fat tissue in the abdomen increases the risk of coronary calcification [45]. Subjects with diabetes and obesity, frequently share deficiency in vitamin D or vitamin K2 which may contribute to high prevalence of vascular calcification [46].

Obesity is associated with increased blood pressure and high levels of leptin. Leptin influences the nitric oxide production and activates the sympathetic system, causing sodium retention, systemic vasoconstriction, and blood pressure elevation. The modulation of leptin’s effects leads to regulation of energy homeostasis to reduce calorie intake and increase energy expenditure which allows to balance blood pressure. So, leptin has a dual function on blood pressure control. Also, the renin–angiotensin–aldosterone system plays an important role in regulating blood pressure and vascular resistance, which influence the cardiac state and arterial pressure [15, 47–50].

Metabolic homeostasis is regulated by incretins, like GLP-1, which are gut hormones released in response to a meal and influence regulation of insulin and the cardiovascular system. GLP-1 stimulates insulin release by modulating the GI functions and control appetite. It is degraded by enzyme dipeptidyl peptidase-4 (DPP-4), involved in adipose tissue inflammation, which in its way is related to insulin resistance [17, 28].

**Fig. 2:**
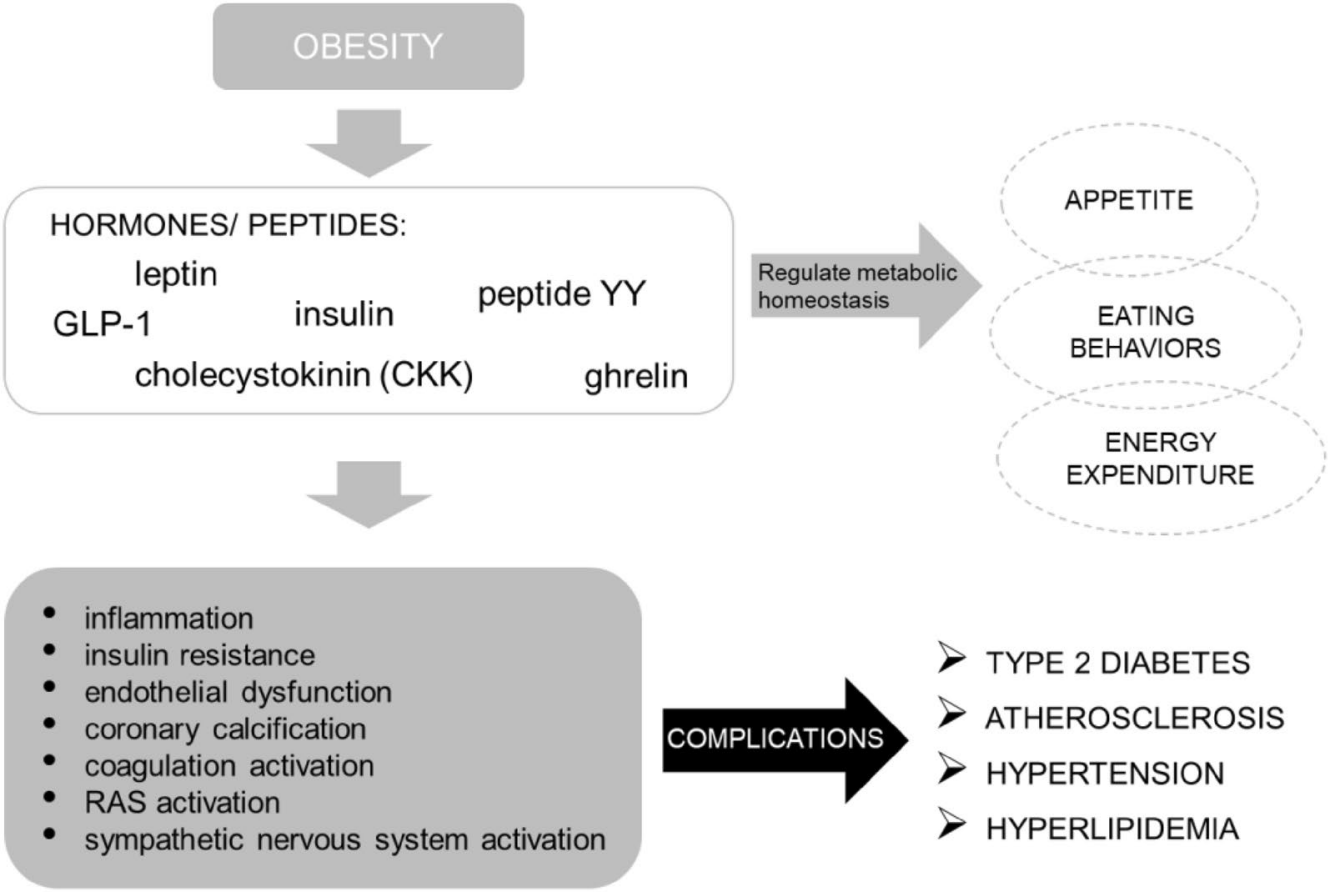
Relationship of obesity and cardiovascular risk. *GLP-1* glucagon-like peptide 1, *RAS* renin angiotensin system

Obesity increases DPP-4 expression [51] reducing the cardiovascular and metabolic effects mediated by GLP-1 levels. This impairment in the incretin axis promotes an imbalance between GLP-1 and GLP-2 which in turn contributes to insulin resistance and dyslipidemia [52]. In addition, in obesity, secretion of GLP-1 is reduced causing an incretin dysregulation and consequently blocking satiety. Whereas DPP-4 either aggravates the incretin defect or stimulates T cell proliferation, increased concentrations have shown to be positively related with BMI, insulin and leptin levels, and negatively associated with adiponectin. These aspects seem relevant in the management of obesity [17, 28] (Fig. 2).

## Metabolically healthy people with obesity

Metabolically healthy people with obesity (MHO) are people with obesity that do not present increased cardiometabolic risk, i.e. the disturbances normally associated to obesity, like insulin resistance, type 2 diabetes, hypertension, and dyslipidemia [53, 54].

There are however, several different criteria used to define metabolic health status. Consequently, the evidence regarding MHO is broad and its prevalence varies widely. Depending on authors and approaches, the factors that determine an individuals’ metabolic health status include blood pressure, triglyceride, cholesterol (total, HDL and LDL), fasting plasma glucose, homeostasis model assessment (HOMA), visceral fat and ectopic fat deposition. In addition to the characterization of obesity that can also be derived from different methods including BMI, waist circumference, and body fat percentage [54].

Hinnouho et al. [53] assessed the risk of mortality among MHO subjects compared to metabolically unhealthy people with obesity (MUO) and metabolically healthy-normal weight subjects, using different criteria. Conclusions have shown that not only MHO subjects did not have a lower risk of mortality (all cause and CVD) compared with the metabolically healthy-normal weight but their risk of mortality was similar to that of the MUO subjects.

So, the criteria and determinants of metabolically healthy obesity remain unclear and need further and better definition to understand the long-term health consequences on this population, whose condition of being MHO changes over time and eventually developing into metabolic and/or mechanical comorbidities.

Finally, another frontier of interest involving obesity and cardiovascular disease is the composition of human microbiota. Obesity seems to be related to cardiovascular events in part due to an imbalance between protective and harmful microbiome, which may determine low-grade inflammation (through the activation of inflammasome), key metabolites such as trimethylamine N-oxide (TMAO), and impairment of the innate immune system [55, 56].

## Available treatments for obesity

In clinical studies, weight loss of around 5–10% can result in a reduced risk of T2D and cardiovascular disease. Anti-obesity medications increase the likelihood of achieving clinically meaningful (≥ 5%) weight loss when used as an adjunct to lifestyle intervention. Pharmacological intervention as adjunct to diet and exercise is indicated for individuals with a BMI ≥ 30 or ≥ 27 kg/m^2^ with at least one obesity-related comorbidity.

The available treatments, currently approved by FDA, EMA and in Brazil, and clinical trials outcomes are described below and summarized in Table 1.

**Table 1:** Clinical trials outcomes studies for anti-obesity agents

Study	Intervention	Population	N enrolled	Design	Primary outcome	Result
XENDOS [59]	Orlistat	Obese	3305	Phase III	Weight loss	2.7 kg orlistat
Phase III [58]	Orlistat	Obese	80	Phase III	Weight loss	4.6 kg orlistat 2.5 kg placebo
Meta-analysis [57, 59]	Orlistat	Overweight or obese	6021	Meta-analysis	Weight loss	2.9% orlistat Reduce blood pressure Reduce LDL cholesterol Reduce fasting glucose
SCALE Obesity and Pre-diabetes [61]	Liraglutide 3.0 mg	Overweight or obese	3731	Phase III	Weight loss	8.4 kg liraglutide 2.8 kg placebo Reduce blood pressure Improvement in fasting lipids, C-reactive protein, plasminogen activator inhibitor-1, adiponectin
SCALE Maintenance [64]	Liraglutide 3.0 mg	Overweight or obese with comorbidities	422	Phase III	Weight loss maintenance	6.2% liraglutide 3.0 mg 0.2% placebo Reduce BMI, waist circumference, glycemic parameters, hsCRP, systolic blood pressure
SCALE Obesity and Pre-diabetes (3-year assessment) [65]	Liraglutide 3.0 mg	Overweight or obese prediabetic with comorbidities	2210	Phase III	Reduce bodyweight and onset of T2D	6.1% liraglutide 3.0 mg 1.9% placebo Reduce BMI, waist circumference, glycemic parameters, systolic blood pressure
Post-hoc MACE-SCALE analysis [66]	Liraglutide 3.0 mg	Overweight or obese	5908	Pooled data	1st occurrence of CV death, nonfatal MI or nonfatal stroke	1.54 CV events/1000 person-years liraglutide 3.0 mg 3.65 CV events/1000 person-years comparator (placebo/orlistat) Reduce blood pressure No increased CV risk
LEADER [67]	Liraglutide 1.8 mg	High-risk population	9340	Phase III	CV safety	13.0% CV events liraglutide 14.9% CV events placebo 4.7% CV deaths liraglutide 6.0% CV deaths placebo
BLOOM [60, 69]	Lorcaserin hydrochloride	Overweight or obese	3182	Phase III	Weight loss	5.8 kg lorcaserin 2.2 kg placebo
BLOSSOM [60, 69]	Lorcaserin hydrochloride	Overweight or obese with comorbid risk factors	4008	Phase III	Weight loss	5.8 kg lorcaserin bid 4.7 kg lorcaserin od 2.9 kg placebo
BLOOM-DM [60, 69]	Lorcaserin hydrochloride	Obese and diabetic	604	Phase III	Weight loss	5.9 kg lorcaserin tid 5.6 kg lorcaserin od 1.9 kg placebo
CAMELLIA-TIMI 61 [68]	Lorcaserin hydrochloride	Overweight or obese at high CV and metabolic risk	12,000	Phase IV	CV safety	38.7% lorcaserin (≥ 5%) 17.4% placebo (≥ 5%) 2.0% CV events/year lorcaserin 2.1% CV events/year placebo 4.1% major CV events/year lorcaserin 4.2% major CV events/year placebo Slightly better values blood pressure, heart rate, glycemic control, lipids More serious hypoglycemia
COR-I [57, 60, 72]	Naltrexone hydrochloride/bupropion hydrochloride	Overweight or obese	1742	Phase III	Weight loss	4.8% NB32 3.7% NB16 Reduce waist circumference, triglycerides, hsCRP Increase HDL cholesterol 1 death due to acute myocardial infarction in NB32 patient 0.2% CV events NB patients 0.3% CV events placebo
COR-II [57, 60, 72]	Naltrexone hydrochloride/bupropion hydrochloride	Overweight or obese	1496	Phase III	Weight loss	5.2% NB32 Reduce waist circumference, triglycerides, hsCRP Increase HDL cholesterol
COR-BMOD [57, 60, 72]	Naltrexone hydrochloride/bupropion hydrochloride	Overweight or obese with controlled hypertension and/or dyslipidemia with or without lifestyle modification	793	Phase III	Weight loss	54.3% NB32 (≥ 5%) 41.6% placebo (≥ 5%) Improvement in hsCRP, fasting blood glucose
COR-Diabetes [60, 72]	Naltrexone hydrochloride/bupropion hydrochloride	Overweight or obese diabetic	505	Phase III	Weight loss	3.2% NB32 No increase of hypoglycemia Decrease in HbA1c
LIGHT Study [57, 73]	Naltrexone hydrochloride/bupropion hydrochloride	Overweight or obese at increased risk	8900	Phase III	MACE	Early terminated
EQUATE [60]	PHEN/TPM	Overweight or obese	776	Phase II	Weight loss	9.2% PHEN/TPM 15/92 8.5% PHEN/TPM 7.5/46 6.4% topiramate 92 mg 6.1% phentermine 15 mg 1.7% placebo
CONQUER [60, 69]	PHEN/TPM	Overweight or obese	2487	Phase III	Weight loss and comorbidities	10.2 kg PHEN/TPM 15/92 8.1 kg PHEN/TPM 7.5/46 1.4 kg placebo Improvement in waist circumference, blood pressure, lipids
EQUIP [69]	PHEN/TPM	Obese	1267	Phase III	Weight loss	10.9% PHEN/TPM 15/92 5.1% PHEN/TPM 3.75/23 1.6% placebo Improvements in fasting blood glucose, blood pressure, cholesterol, waist circumference in PHEN/TPM ER 15/92 mg
AQCLAIM	PHEN/TPM	Overweight or obese with documented CVD	Target 540	Phase III	Time to 1st occurrence of nonfatal MI, nonfatal stroke or CV death	On going
SCOUT [74]	Sibutramine	Overweight or obese + 55-year at high CV risk	10,744		CV events	− 1.7 kg (1 year) sibutramine + 0.7 kg (1 year) placebo Higher risk of a primary outcome event, nonfatal myocardial infarction Increased blood pressure, pulse rate, cardiovascular events (tachycardia, hypertension, arrhythmias)
Meta-analysis [59]	Sibutramine	Overweight or obese	929	Phase III	Weight loss	4.6% (1 year)
Phase III [75]	Sibutramine	Obese	224	Phase III	Weight loss	5.0 kg (1 year)

*bid* twice daily, *BMI* body mass index, *CV* cardiovascular, *hsCRP* high-sensitivity C-reactive protein, HDL high density lipoprotein, *LDL* low density lipoprotein, *MACE* major adverse cardiovascular events, *MI* myocardial infarction, *NB32* naltrexone (32 mg/day) + bupropion (360 mg/day) in a fixed-dose formulation, od once a day, *PHEN/TPM* Phentermine hydrochloride/topiramate, *T2D* type 2 diabetes

### Orlistat

Orlistat, or tetrahydrolipstatin, is a pancreatic and gastric selective lipase inhibitor, approved as an anti‐obesity drug [57, 58]. It is prescribed orally at therapeutic doses of 120 mg three times daily (tid) with meals, administered with a well-balanced diet. Its activity is dose-dependent with approximately 30% inhibition of dietary fat absorption and the major route of elimination is fecal excretion [57]. Orlistat is approved in the US, Europe, and other countries, like Brazil.

Data from published clinical trials with orlistat for the treatment of obesity include a 4-year, double blind, placebo-controlled, randomized study in 3305 Swedish people with obesity (XENDOS study) [59] where orlistat reduced weight by 2.7 kg on average. In another study [58], a 24-week prospective, randomized, single blind study between orlistat (120 mg three times a day) and placebo in 80 adult people with obesity, orlistat has shown a reduction in weight (4.65 kg vs 2.5 kg in placebo), BMI (1.91 kg/m^2^ vs 0.64 kg/m^2^), waist circumference (4.84 cm vs 2.00 cm), cholesterol and LDL level, when compared to placebo. Also, in a meta-analysis of 11 placebo-controlled trials of 1 year in 6021 people with overweight or people with obesity, orlistat reduced weight by 2.9% and the number of patients with 5% and 10% placebo success in weight-loss was 21% and 12% greater with orlistat than with placebo. In this meta-analysis, orlistat also reduced blood pressure, LDL cholesterol and fasting glucose in patients with diabetes [57, 59]. Efficacy of orlistat has been demonstrated in diverse group of people with obesity including adolescent, adults with metabolic syndrome, pre-diabetics, type 2 diabetic [58].

The major adverse effects with orlistat reported in all studies are gastrointestinal. Loose stools, oily stools/spotting, abdominal pain and fecal urgency were observed in 15–30% of orlistat-treated patients and 2–7% in placebo [58, 59]. Orlistat did not produce any adverse impact on the Hb, total leukocyte count (TLC), serum creatinine, SGPT and SGOT [58]. Orlistat has proven to be a well-tolerated anti-obesity drug, to be used additionally to dietary and lifestyle changes [57–59] (Table 1).

### Liraglutide

Liraglutide is a long-acting human glucagon like peptide-1 (GLP-1) analogue receptor agonist approved for chronic weight management in patients with a BMI ≥ 27 kg/m^2^ and a weight related comorbid condition [60, 61]. Liraglutide has been shown to directly stimulate pro-opiomelanocortin (POMC) neurons and inhibit neuropeptide-Y and agouti-related peptide neurons of the arcuate nucleus resulting in appetite suppression [62]. These actions may also be accompanied by effects on other areas of the brain such as the mesolimbic system resulting in diminished food-induced reward signals. Liraglutide alters brain activity related to highly desirable food cues [63].

Liraglutide is administered subcutaneously as an isotonic solution with peak absorption at 11 h after injection and absolute bioavailability of 55%. Weight loss with liraglutide is dose-dependent up to 3.0 mg once daily (od) and is mediated by hypothalamic action in neurons involved in the energetic balance, and by reduced appetite and energy intake rather than by increased energy expenditure [60, 61]. Liraglutide 3.0 mg has received regulatory approval for weight management in adults in the US, Europe, and other countries, like Brazil.

In the SCALE Obesity and Pre diabetes study, a 56-week, randomized, double-blind, placebo-controlled clinical trial [61] in 3731 people with overweight or obesity without type 2 diabetes, patients treated with liraglutide lost a mean of 8.4 ± 7.3 kg of body weight versus 2.8 ± 6.5 kg in placebo (a difference of − 5.6 kg). In this study, 63.2% of patients in the liraglutide group lost at least 5% of their body weight and 33.1% lost more than 10% of their body weight, with statistical significance when compared to placebo (respectively 27.1% and 10.6%). Also, results for blood pressure were lower in the liraglutide group while measures of fasting lipid levels, C-reactive protein, plasminogen activator inhibitor-1, and adiponectin exhibited greater improvement also in the liraglutide group when compared to placebo. Liraglutide (3.0 mg) is considered safe and, as an adjunct to diet and exercise, has proven to reduced body weight in clinically meaningful manner and improve metabolic control [61].

The SCALE Maintenance study [64], a randomized, 56-week, phase 3 clinical trial evaluated the efficacy of liraglutide 3.0 mg/day or placebo in maintaining weight loss achieved with a low-calorie diet in people with overweight/obesity with comorbidities. The 422 patients included had a mean weight loss of 6.0% during the run-in period. At week 56, weight change from randomization has shown an additional mean weight loss of 6.2% for liraglutide (in a total of 12.2% weight loss) and 0.2% for placebo, with a statistically significant difference of 6.1% (4.6%–7.5%). Maintenance of weight loss (≥ 5%) was more evident in patients treated with liraglutide than with placebo, both compared with run-in period (81.4% vs 48.9% in placebo) or randomization (50.5% vs 21.8%). At week 56, post randomization, significantly greater decreases were seen in liraglutide patients for BMI, waist circumference, glycemic parameters, high-sensitivity C-reactive protein and systolic blood pressure.

An extension of the SCALE Obesity and Pre-diabetes study [65], continued screened patient for a further 2 years to a the 3-year assessment, placebo-controlled trial in people with obesity or overweight prediabetic adults with comorbidities. The effect of liraglutide 3.0 mg was evaluated as an adjunct to a reduced-calorie diet and increased physical activity in delaying time to onset of T2D, as well as weight loss and safety over 3 years. Results for 2210 patients showed that liraglutide induced greater weight loss than placebo (− 6.1% vs − 1.9%) with and estimated treatment, statistically significant, difference of 4.3%. Approximately 25% of liraglutide treated patients and 10% of placebo patients lost more than 10% of bodyweight. Significantly greater decreases were seen in liraglutide patients for BMI (− 2.4 vs − 0.7 kg/m^2^), waist circumference (− 6.9 vs − 3.4 cm), glycemic parameters (glycated hemoglobin, fasting glucose, fasting insulin), and systolic blood pressure (− 3.2 vs − 0.5 mmHg).

A post hoc analysis [66] of 5 randomized, double-blind clinical trials evaluated the cardiovascular safety of liraglutide 3.0 mg in 5908 patients versus a comparator group (placebo or orlistat). The primary outcome of this analysis was the first occurrence of cardiovascular death, nonfatal myocardial infarction or nonfatal stroke, and the cardiovascular events were adjudicated prospectively for three of the trials and retrospectively for the other two. Results have shown that 8 patients treated with liraglutide 3.0 mg had cardiovascular events (1.54 events/1000 person-years) and 10 patients in the comparator group (3.65 events/1000 person-years). The hazard ratio was 0.42 (95% CI 0.17–1.08), with liraglutide not being associated with an increased rate of cardiovascular events as compared with the comparators. In this analysis, liraglutide was associated with significantly reduced mean systolic and diastolic blood pressure compared to placebo returning to baseline values upon treatment discontinuation. These results suggest no increased risk of liraglutide 3.0 mg on cardiovascular safety and a possible benefit in populations of people with overweight/obesity.

In all SCALE phase III studies (SCALE Obesity and Pre-diabetes 1 and 3-year assessment, SCALE Maintenance, SCALE Sleep apnea, SCALE Diabetes) the most common adverse events reported were nausea, diarrhea and constipation in transient and mild/moderate intensity, with higher incidence in the liraglutide treated patients [61, 64–66].

These observations are in agreement with the LEADER trial results, a large trial testing the cardiovascular safety of liraglutide 1.8 mg among T2D high-risk population confirming better metabolic profile and reduced cardiovascular and all-cause mortality [67] (Table 1).

### Lorcaserin hydrochloride

Lorcaserin HCl is a small-molecule agonist of the serotonin 2C (5-HT2C) receptor indicated for people with overweight or obesity as an adjunct to a reduced-calorie diet and increased physical activity and at least one weight-related comorbidity (i.e. hypertension, dyslipidemia, type 2 diabetes) [68, 69]. It acts selectively at 5-HT receptors in the hypothalamus by stimulation of satiety centers, activating the anorexigenic POMC pathway, to reduce appetite. With a functional selectivity of 15 times higher affinity for 5-HT2C than for 5-HT2A receptors and 100 times higher selectivity for 5-HT2C than for the 5-HT2B receptors, seen as an advantage to both its efficacy and safety since its appetite-suppressing effects act while avoiding cardiovascular effects which are usual in other nonselective serotonergic weight-loss medications [60, 68]. Lorcaserin’s binding affinity is dose-dependent at the maximum dose of 20 mg/day. Lorcaserin is approved in the US and other countries, like Brazil (although not commercialized in Brazil).

The BLOOM trial (Behavioral Modification and Lorcaserin for Overweight and Obesity Management), a double blinded, randomized, placebo-controlled study in 3182 adults with obesity or overweight evaluated weight loss in patients treated with lorcaserin HCl 10 mg twice daily (bid)or placebo for 2 years. After 1 year of treatment, patients treated with lorcaserin HCl lost an average of 5.8 kg versus 2.2 kg in placebo, which corresponds to 47.5% of patients in lorcaserin versus 20.3% of patients in placebo with ≥ 5% weight-loss, with statistical significance. Approximately 22% of patients in the lorcaserin group and 7% of patients in the placebo group achieved ≥ 10% weight-loss with statistical significance. Lorcaserin was overall well tolerated [60, 69].

The BLOSSOM trial was a double-blind, randomized, placebo-controlled study with 4008 adults with overweight or obesity with comorbid risk factors. Patients were treated with lorcaserin HCl 10 mg twice daily, lorcaserin HCl 10 mg once daily or placebo in addition to counseling on diet and exercise for 1 year. Patients under lorcaserin twice daily had a 5.8 kg weight loss (47.2% with ≥ 5% weight loss) while patients on lorcaserin once daily lost 4.7 kg (40.2% with ≥ 5% weight loss) and patients on placebo lost 2.9 kg (25% with ≥ 5% weight loss). The proportion of patients achieving 10% total weight loss were 22.6% in lorcaserin twice daily, 17.4% in lorcaserin once daily, and 9.7% in placebo [60, 69].

The BLOOM-DM trial was an extension of the BLOOM trial but in people with obesity and diabetic patients. The study included 604 adults with obesity with type 2 diabetes who were treated with metformin, a sulfonylurea, or both and were assigned to receive lorcaserin HCl 10 mg twice daily, lorcaserin HCl 10 mg once daily or placebo for 1 year. Registered weight loss observed was of 5.9 kg in the twice-daily lorcaserin, 5.6 kg in the once-daily lorcaserin patients and 1.9 kg in the placebo group. The adverse events reported were similar to previous trials (headache, nausea, back pain, upper respiratory infection, dizziness and fatigue, more frequent in patients receiving lorcaserin), however symptomatic hypoglycemia was observed and more common in patients treated with lorcaserin HCl (8.4% vs 6.3% in placebo). There was no difference in valvulopathy between the groups both in the BLOOM and BLOSSOOM trials, however, in the BLOOM-DM trial a non-statistically significant new valvulopathy occurred in 0.5% of patients in placebo, in 2.5% once-daily lorcaserin and in 2.9% twice-daily lorcaserin patients [60, 69].

The CAMELLIA-TIMI 61 (Cardiovascular and metabolic effects of lorcaserin in overweight and obese patients-thrombolysis in myocardial infarction 61) trial [68], a randomized, double-blind, placebo-controlled, multinational study was designed to evaluate efficacy and long-term cardiovascular safety of lorcaserin in patients with overweight or obesity at high cardiovascular and metabolic risk. Twelve thousand patients in 8 countries were randomized to receive lorcaserin 10 mg twice daily or placebo for 5 years. After 1 year of treatment, 38.7% of patients in the lorcaserin group and 17.4% in placebo had weight loss ≥ 5% (p < 0.001). Major cardiovascular events like cardiovascular death, myocardial infarction or stroke, in a median follow-up of 3.3 years, occurred in 2.0% per year in the lorcaserin and in 2.1% per year in the placebo group. The extended major cardiovascular events such as heart failure, or hospitalization for unstable angina or revascularization, occurred in 4.1% per year in the lorcaserin and in 4.2% per year in the placebo group. For cardiac risk factors (blood pressure, heart rate, glycemic control, and lipids) patients treated with lorcaserin had slightly better values than those in placebo. Adverse events were similar in the two groups, however patients in the lorcaserin group reported more serious hypoglycemia. The overall results of this trial suggested cardiovascular safety in a large high-risk population of subjects with overweight or obesity [70] (Table 1).

### Naltrexone hydrochloride/bupropion hydrochloride

The naltrexone-bupropion combination pill is a sustained-release formulation of two centrally acting medications composed of 8 mg of naltrexone and 90 mg bupropion. The mechanism of action of naltrexone-bupropion is a combination of both medications. Pro-opiomelanocortin-producing neurons in the hypothalamus release α-melanocyte-stimulating hormone (MSH) and β-endorphin. α-MSH mediates the anorectic effect of POMC, whereas β-endorphin is responsible for autoinhibitory feedback, which inactivates the anorectic effect. Bupropion can be used to stimulate the POMC neurons, whereas naltrexone can be used to block the autoinhibitory feedback that is associated with a decline in weight reduction [57, 60, 71].

It is approved in the USA and Europe for long-term weight management in patients with obesity and obesity-related comorbidities, in addition to caloric restrictions and lifestyle intervention. The recommended total daily dose is 32 mg naltrexone and 360 mg bupropion that should be initiated with one tablet of 8 mg naltrexone/90 mg bupropion a day, increased over 3 weeks to the maintenance dose of two tablets of 8 mg/90 mg twice a day. The 32 mg of naltrexone is the optimum dose [57, 60, 71, 72]. In the beginning of the treatment nausea is frequently reported, and seizures, elevated blood pressure or myocardial infarction are other side effects that have been observed [60].

Four phase III studies evaluated the efficacy and safety of the naltrexone-bupropion combination versus placebo, for 56 weeks. COR-I study randomized 1742 patients to either naltrexone (16 mg/day) + bupropion (360 mg/day) in a fixed-dose formulation (NB16), naltrexone (32 mg/day) + bupropion (360 mg/day) in a fixed-dose formulation (NB32) or placebo. The mean weight loss was 4.8% for NB32 group and 3.7% for NB16. Weight loss ≥ 5% was 48% for NB32, 39% for NB16 and 16% for placebo, with a statistically significant difference between NB16 and NB32. NB 16 and NB 32 showed significant improvements in waist circumference, triglycerides, high-sensitivity C-reactive protein (hsCRP) and HDL cholesterol levels over placebo [57, 60, 72].

COR-II evaluated 1496 patients assigned to NB32 (1001) or placebo (496) for 56 weeks and the results were similar to COR-I, with a mean weight loss of 5.2% for NB32 and weight loss ≥ 5% of 50.5% for NB32 and 17.1% for placebo. The most common adverse event in both COR trials was nausea, which occurred 2–3 times as much in treatment group (5.3%-10.5%) when compared to placebo (29.2%–42.3%), being transient in the first weeks of treatment. Other events reported included headache, dizziness, insomnia and vomiting. Constipation, upper abdominal pain and migraine were reported as severe and more frequent in NB group [57, 60, 72].

COR-BMOD assessed safety and efficacy in 793 patients with overweight or obesity with controlled hypertension and/or dyslipidemia with or without lifestyle modification over 56 weeks. Due to lifestyle modification, the placebo group lost more weight than reported in previous studies, with 41.6% of patients in placebo achieving ≥ 5% weight loss compared to 54.3% of NB32 patients. NB32 patients showed significant improvements in hsCRP and fasting blood glucose values. Two patients in the NB32 group reported cholecystitis as serious adverse event [57, 60, 72].

In COR-Diabetes study which enrolled 505 patients with overweight or obesity with type 2 diabetes, the NB32 group of treatment had a weight loss of 3.2% with 44.5% of patients with ≥ 5% weight loss compared to 18.9% in placebo [60, 72].

There was one death by acute myocardial infarction in a NB32 patient in COR-I, even though overall, the incidence of cardiovascular events was low, 0.2% in NB patients and 0.3% in placebo patients [60].

Another phase III study (the LIGHT Study [57, 73]) randomized, double-blind, placebo-controlled, to assess the occurrence of major adverse cardiovascular events (MACE) in 8900 patients with overweight or obesity at increased risk treated with an NB32 or placebo. Interim analyses for this study performed after 25% and 50% of planned events, showed that the hazard ratio for MACE did not exceed 2.0 for NB32 compared to placebo. However, this trial was terminated early and it was not possible to assess noninferiority of NB over placebo [73] (Table 1).

### Phentermine hydrochloride/topiramate (PHEN/TPM)

The combination of immediate-release phentermine with extended-release topiramate in one pill, is an amphetamine analog stimulant designed for the short-term treatment of obesity in adults with obesity or overweight, with at least one obesity-related condition, and in addition to a low-calorie diet and increased physical activity. PHEN/TPM acts through the two mechanisms of action of its components. Phentermine antagonizes alpha-adrenergic receptors, like norepinephrine which in response are released into the hypothalamus, resulting in an increase in blood leptin concentration and appetite suppression. Topiramate increases the activity of neurotransmitter gamma-aminobutyric acid (GABA), to modulate voltage-gated ion channels and inhibit carbonic anhydrase or AMPA/kainite excitatory glutamate receptors [69]. Phentermine/topiramate is administered once a day in doses varying from low (3.75/23 mg), intermediate (7.5/46 mg) or high (15/92 mg). PHEN/TPM has potential teratogenic risk, as well as cardiovascular risk with increase of the heart rate [60]. Phentermine/topiramate at the top dose is one of the most effective pharmacotherapies for the treatment of obesity in the US market, but it is not available for prescription in European or Brazilian markets.

One phase II and two phase III studies have been published with efficacy results for the combination phentermine/topiramate. The EQUATE trial [60], a phase II study evaluated 776 patients randomized to phentermine monotherapy (7.5 and 15 mg), topiramate monotherapy (46 and 92 mg), PHEN/TPM 7.5/46, PHEN/TPM 15/92 or placebo for 28 weeks. Patients under the combination PHEN/TPM both 15/92 and 7.5/46 have shown a higher proportion of weight loss at the end of study: 9.2% in PHEN/TPM 15/92, 8.5% in PHEN/TPM 7.5/46, 6.4% in topiramate 92 mg, 6.1% in phentermine 15 mg and 1.7% in placebo.

The CONQUER trial, a randomized, double-blind, placebo-controlled phase 3 study included 2487 adult patients with overweight -or obesity patients to evaluate the effect of PHEN/TPM 7.5/46, PHEN/TPM 15/92 or placebo on weight and associated comorbidities over 56 weeks. Results favored PHEN/TPM with weight loss of 8.1 kg for PHEN/TPM ER 7.5/46 and 10.2 kg for PHEN/TPM ER 15/92 mg, compared to 1.4 kg for placebo, with statistical significance. Overall, 62% of the patients treated with PHEN/TPM ER 7.5/46 and 70% in PHEN/TPM ER 15/92 achieved the goal of ≥ 5% weight loss versus 21% of patients in placebo [60, 69]. Most markers for cardiovascular risk, such as waist circumference, blood pressure and lipids, have shown an overall significant improvement. Side effects commonly reported were dry mouth, paresthesia, flu, upper respiratory infection, change in taste and insomnia [60].

The EQUIP trial [69] was a double-blind, parallel-group study in 1267 adult patients with obesity assigned to either PHEN/TPM ER 3.75/23 mg, PHEN/TPM ER 15/92 mg or placebo for 56 weeks. All treatment groups had statistically significant weight loss at the end of the 56 weeks, respectively 10.9% for PHEN/TPM ER 15/92 mg, 5.1% for PHEN/TPM ER 3.75/23-mg and 1.6% for placebo. Patients treated with PHEN/TPM ER 15/92 mg have shown the better results in achieving improvements in the obesity-related complications fasting blood glucose, blood pressure, cholesterol and waist circumference.

The AQCLAIM study, and event-driven, randomized, double-blind, placebo-controlled study to evaluate cardiovascular morbidity and mortality in patients with documented cardiovascular disease. This study is ongoing with the primary efficacy endpoint as time to first occurrence of a primary outcome event (nonfatal MI, nonfatal stroke, or cardiovascular death) (Table 1).

### Sibutramine

Sibutramine is a β-phenethylamine or a selective norepinephrine and serotonin reuptake inhibitor that increase the levels of endogenous catecholamines and acts centrally to increase satiety. It was approved for weight management in patients unable to lose weight with diet and physical activity alone. Sibutramine induces satiety and an increase in energy expenditure, however it also increases blood pressure, pulse rate, or both due to its sympathomimetic effects, therefore is not indicated for patients with history of cardiovascular disease [59, 74]. The drug was approved in the US market in 1997 and in Europe in 2001, however due to increased cardiovascular events in 2010 it has been withdrawn from the market in several countries and regions of the world including the European Union and the United States. Nevertheless, the drug remains available in Brazil.

In three randomized, double-blind, placebo-controlled clinical trials, sibutramine has reduced weight on average by 4.6% in 929 people with overweight or obesity over 1 year. Between 28% and 40% of patients achieved the 5% placebo-subtracted weight-loss and 4% to 27% achieved the 10% goal, favoring sibutramine over placebo [59].

A randomized trial in 224 adults with obesity treated with sibutramine alone, sibutramine + brief individualized lifestyle modification, group lifestyle modification alone or sibutramine + group lifestyle modification during 1-year has shown that more weight was lost on the sibutramine + lifestyle modification (average of 12.1 kg) compared with sibutramine alone (5.0 kg) [75].

The long-term effects of sibutramine treatment on the rates of cardiovascular events and cardiovascular death among + 55-year adults with overweight or obesity at high cardiovascular risk was determined in the SCOUT trial [74]. This was a randomized, double-blind, placebo-controlled, multicenter trial conducted between 2003 and 2009 in 16 countries all over the world (Europe, Central America, South America and Australia). Having history of cardiovascular disease (coronary artery disease, stroke, or peripheral arterial occlusive disease), and/or type 2 diabetes with cardiovascular risk factor (hypertension, dyslipidemia, current smoking or diabetic nephropathy) was required to all 10,744 patients enrolled to receive sibutramine or placebo combined with a weight-management program (diet and exercise). The mean duration of treatment was 3.4 years. After randomization, the sibutramine patients showed a mean weight reduction of 1.7 kg at 12 months, while the placebo group patients had a mean weight increase of 0.7 kg. Patients treated with sibutramine have shown a higher risk of a primary outcome event (11.4% versus 10.0% in the placebo group as well as risk of nonfatal myocardial infarction (4.1% versus 3.2% in placebo) and nonfatal stroke (2.6% versus 1.9% in placebo), but not of cardiovascular death or death from any cause.

Side-effects most commonly associated to sibutramine include insomnia, nausea, dry mouth and constipation. Also, sibutramine has been associated with increased blood pressure and pulse rate, and some cardiovascular events, like tachycardia, hypertension and arrhythmias. For this reason, this drug is not recommended in patients with uncontrolled hypertension, pre-existing cardiovascular disease or tachycardia, and ultimately has been withdrawn from the market in several countries in 2010 [59] (Table 1).

## Conclusions

Obesity is a chronic, multifactorial disease with a complex pathogenesis and associated difficulties in attaining goals, maintenance objectives and satisfactory outcomes (just with nutritional education or physical activity) with lifestyle adjustments and treatment success. Pharmacological interventions help controlling the disease but are not always successful in the long term, even though the results are better when associated to lifestyle changes. In order to accomplish satisfactory goals, patients and physicians seek for weight loss, weight maintenance and improvement of the risk factors associated to this condition, especially cardiovascular risk.

We here present an overview of the intrinsic mechanisms of obesity and its relation to cardiovascular risks, and the available treatments to overcome this condition which should be managed by a multidisciplinary team.

## Data Availability

Not applicable.
